# Supplementary material for: Genome comparisons indicate recent transfer of wRi‐like Wolbachia between sister species Drosophila suzukii and D. subpulchrella
Source: Ecol Evol. 2017 Oct 8;7(22):9391–404. doi: 10.1002/ece3.3449 (PMC5696437; doi:10.1002/ece3.3449)
Supplement: Supplementary file 1 [file ECE3-7-9391-s001.docx]

| Table S1 Near-universal, single-copy proteobacteria genes (out of 221) found using BUSCO v. 3.0.0. | | | |
| --- | --- | --- | --- |
| Genome | Complete, single copy | Fragment | Not found |
| *w*Ri | 180 | 2 | 39 |
| *w*Mel | 180 | 2 | 39 |
| *w*Au | 181 | 2 | 38 |
| *w*Ha | 179 | 3 | 39 |
| *w*No | 181 | 4 | 36 |
| *w*Suz | 182**^*^** | 2^†^ | 37 |
| *w*Spc | 182**^*^** | 2^†^ | 37 |

**^*^**Ribonuclease H-like domain is complete in *w*Suz and *w*Spc, but was not found in *w*Ri, *w*Mel, *w*Au, *w*Ha or *w*No. Dihydropteroate synthase is complete in *w*Suz, *w*Spc, *w*Au and *w*No, but was not found in *w*Ha, *w*Mel or *w*Ri.

^†^Peptidyl-tRNA hydrolase is complete in *w*Suz, *w*Spc, and *w*No, but is fragmented in *w*Ri, *w*Au, *w*Ha and *w*Mel. Phospho-N-acetylmuramoyl-pentapeptide-transferase is fragmented in *w*Suz, *w*Spc and *w*Ha, but is complete in *w*Au, *w*Mel and *w*No.

| Table S2 Observed pairwise genomic differences between *Wolbachia* strains, given as percentage of polymorphic sites in single-copy, full-length genes present in all strains. | | | | |
| --- | --- | --- | --- | --- |
| *w*Spc-*w*Suz-*w*Ri dataset (704,883 base pairs, 703 genes) | | | | |
| Genomes | | **Number of differences** | **Percent difference** | |
| *w*Spc v. *w*Suz | | 28 | 0.004% | |
| *w*Ri v. *w*Suz | | 103 | 0.014% | |
| *w*Ri v. *w*Spc | | 99 | 0.015% | |
| *w*Spc-*w*Suz-*w*Ri-*w*Mel-*w*Au dataset (480,831 base pairs, 512 genes) | | | | |
| Genomes | **Number of differences** | | | **Percent difference** |
| *w*Spc v. *w*Suz | 21 | | | 0.005% |
| *w*Suz v. *w*Ri | 62 | | | 0.014% |
| *w*Spc v. *w*Ri | 59 | | | 0.014% |

| Table S3 Matrix of *k*_a_ (below diagonal) and *k*_s_ (above diagonal) estimates for *w*Suz, *w*Spc, *w*Ri, *w*Au and *w*Mel (using the 480,831 bp data set from Table S2). | | | | | |
| --- | --- | --- | --- | --- | --- |
|  | ***w*Suz** | ***w*Spc** | ***w*Ri** | ***w*Au** | ***w*Mel** |
| *w*Suz |  | 0.002% | 0.017% | 4.58% | 4.58% |
| *w*Spc | 0.005% |  | 0.019% | 4.57% | 4.58% |
| *w*Ri | 0.013% | 0.014% |  | 4.57% | 4.58% |
| *w*Au | 0.78% | 0.77% | 0.77% |  | 0.20% |
| *w*Mel | 0.78% | 0.78% | 0.78% | 0.10% |  |

| Table S4 The 28 substitutions differentiating *w*Spc and *w*Suz. | | | | |
| --- | --- | --- | --- | --- |
| Gene | **Amino Acid** | ***w*Spc** **codon** | ***w*Suz codon** | **Gene Description** |
| *WRi_000230* | 228 | GTT(Val) | ATT(Ile) | DNA-directed RNA polymerase beta subunit |
| *WRi_000410* | 67 | GCT(Ala) | GCC(Ala) | hypothetical protein |
| *WRi_000410* | 118 | TCG(Ser) | TTG(Leu) | hypothetical protein |
| *WRi_000780* | 310 | TGC(Cys) | TGT(Cys) | GTP/ATP binding protein putative |
| *WRi_001670* | 103 | GAC(Asp) | GAT(Asp) | enoyl-(acyl-carrier-protein) reductase |
| *WRi_002520* | 228 | GAT(Asp) | CAT(His) | GTP-binding protein |
| *WRi_002650* | 6 | GAA(Glu) | AAA(Lys) | hypothetical protein |
| *WRi_003080* | 374 | ACA(Thr) | ACG(Thr) | succinate dehydrogenase flavoprotein subunit |
| *WRi_003240* | 59 | TCT(Ser) | CCT(Pro) | hypothetical protein |
| *WRi_003580* | 722 | TTA(Leu) | CTA(Leu) | hypothetical protein |
| *WRi_004080* | 303 | CGG(Arg) | TGG(Trp) | bicyclomycin resistance protein |
| *WRi_004790* | 793 | CCT(Pro) | TCT(Ser) | hypothetical protein |
| *WRi_004810* | 31 | AAT(Asn) | ACT(Thr) | protoheme IX farnesyltransferase |
| *WRi_006490* | 372 | AAC(Asn) | GAC(Asp) | deoxyguanosinetriphosphate triphosphohydrolase |
| *WRi_006610* | 26 | CAA(Gly) | CCA(Pro) | polysaccharide deacetylase putative |
| *WRi_007380* | 335 | AAT(Asn) | AGT(Ser) | peptidase M16 family |
| *WRi_007510* | 47 | GGG(Gly) | GCG(Ala) | hypothetical protein |
| *WRi_008460* | 296 | TAT(Tyr) | CAT(His) | iron compound ABC transporter periplasmic iron compound-binding protein |
| *WRi_008830* | 45 | ACT(Thr) | GCT(Ala) | hypothetical protein |
| *WRi_008830* | 29 | GCA(Ala) | GTA(Val) | hypothetical protein |
| *WRi_010700* | 179 | ATT(Ile) | ATG(Met) | permease putative |
| *WRi_010800* | 415 | TTG(Leu) | TTT(Phe) | sodium/alanine symporter family protein |
| *WRi_010800* | 226 | ATG(Met) | ATT(Ile) | sodium/alanine symporter family protein |
| *WRi_011150* | 29 | GAA(Glu) | GCA(Ala) | putative monovalent cation/H+ antiporter subunit D |
| *WRi_011880* | 231 | GAT(Asp) | AAT(Asn) | Succinyl-CoA synthetase beta subunit |
| *WRi_012790* | 260 | GGG(Gly) | GAG(Glu) | Type IV secretion system protein VirB9 putative |
| *WRi_012830* | 128 | ATT(Ile) | ATG(Met) | rod shape-determining protein RodA |
| *WRi_012980* | 22 | TGT(Cys) | TTT(Phe) | HIT family protein |

| **Table S5** Genes present in CNV regions of *w*Suz or *w*Spc relative to *w*Ri. All locations are relative to the *w*Ri reference sequence of Klasson *et al*. (2009). | | | | | | | | | |  |
| --- | --- | --- | --- | --- | --- | --- | --- | --- | --- | --- |
| **CNV Location** | | **Copy number change** | | | **Affected genomes** | | | **Kolmogorov-Smirnov P-value** | |  |
| 570000-592500, 1077500-1100000 | | 2 → 3 | | | *w*Suz only | | | <0.0001 | |  |
| **Gene start** | **Gene end** | | | **Gene ID** | | **Gene Description** | | | | |
| 571723 | 573147 | | | *WRi_005370* | | Hypothetical protein: ortholog to *WD0631*, one of the tandem putative CI loci in wMel. | | | | |
| 573202 | 576723 | | | *WRi_p005380* | | Putative pseudogene, a truncated ortholog of *WD0632* (and *wPip_0283*) | | | | |
| 577843 | 580743 | | | *WRi_005390* | | Ankyrin repeat domain protein | | | | |
| 581150 | 582643 | | | *WRi_005400* | | site-specific recombinase resolvase family | | | | |
| 582831 | 583663 | | | *WRi_005420* | | transposase | | | | |
| 584009 | 584467 | | | *WRi_005440* | | ankyrin repeat domain protein | | | | |
| 584493 | 585227 | | | *WRi_005450* | | ankyrin repeat domain protein | | | | |
| 585395 | 586555 | | | *WRi_005460* | | hypothetical protein | | | | |
| 586555 | 587346 | | | *WRi_005470* | | baseplate assembly protein J putative | | | | |
| 587349 | 587684 | | | *WRi_005480* | | baseplate assembly protein W putative | | | | |
| 587687 | 587941 | | | *WRi_005490* | | hypothetical protein | | | | |
| 587949 | 588413 | | | *WRi_005500* | | baseplate assembly protein V | | | | |
| 588400 | 588876 | | | *WRi_005510* | | hypothetical protein | | | | |
| 588873 | 589394 | | | *WRi_005520* | | minor tail protein Z putative | | | | |
| 589396 | 589701 | | | *WRi_005530* | | hypothetical protein | | | | |
| 589799 | 590803 | | | *WRi_005540* | | hypothetical protein | | | | |
| 590841 | 591212 | | | *WRi_005550* | | hypothetical protein | | | | |
| 591287 | 592348 | | | *WRi_005560* | | minor capsid protein C putative | | | | |
| 1078182 | 1079606 | | | *WRi_010030* | | Hypothetical protein: ortholog to *WD0631*, one of the tandem putative CI loci in wMel. | | | | |
| 1079661 | 1083182 | | | *WRi_p010040* | | Putative pseudogene, a truncated ortholog of *WD0632* (and *wPip_0283*) | | | | |
| 1084302 | 1087202 | | | *WRi_010050* | | Ankyrin repeat domain protein | | | | |
| 1087609 | 1089102 | | | *WRi_010060* | | site-specific recombinase resolvase family | | | | |
| 1089290 | 1090122 | | | *WRi_010080* | | transposase | | | | |
| 1090468 | 1090926 | | | *WRi_010100* | | ankyrin repeat domain protein | | | | |
| 1090952 | 1091686 | | | *WRi_010110* | | ankyrin repeat domain protein | | | | |
| 1091854 | 1093014 | | | *WRi_010120* | | hypothetical protein | | | | |
| 1093014 | 1093805 | | | *WRi_010130* | | baseplate assembly protein J putative | | | | |
| 1093808 | 1094143 | | | *WRi_010140* | | baseplate assembly protein W putative | | | | |
| 1094146 | 1094400 | | | *WRi_010150* | | hypothetical protein | | | | |
| 1094408 | 1094872 | | | *WRi_010160* | | baseplate assembly protein V | | | | |
| 1094859 | 1095335 | | | *WRi_010170* | | hypothetical protein | | | | |
| 1095332 | 1095853 | | | *WRi_010180* | | minor tail protein Z putative | | | | |
| 1095855 | 1096160 | | | *WRi_010190* | | hypothetical protein | | | | |
| 1096258 | 1097262 | | | *WRi_010200* | | hypothetical protein | | | | |
| 1097300 | 1097671 | | | *WRi_010210* | | hypothetical protein | | | | |
| 1097746 | 1098807 | | | *WRi_010220* | | minor capsid protein C putative | | | | |
| **CNV Location** | | | **Copy number change** | | | | **Affected genomes** | | **Kolmogorov-Smirnov *P*-value** |  |
| 733000-756000 | | | 1 → 0 | | | | *w*Suz and *w*Spc | | <0.0001 |  |
| **Gene start** | **Gene end** | | | **Gene ID** | | **Gene Description** | | | | |
| 733007 | 734389 | | | *WRi_006770* | | transposase | | | | |
| 735447 | 736526 | | | *WRi_006790* | | hypothetical protein | | | | |
| 736739 | 737194 | | | *WRi_006800* | | Small heat shock protein | | | | |
| 737637 | 738647 | | | *WRi_006810* | | ankyrin repeat domain protein | | | | |
| 738683 | 739515 | | | *WRi_006820* | | transposase IS5 family | | | | |
| 741777 | 749201 | | | *WRi_006850* | | ankyrin repeat domain protein | | | | |
| 749574 | 750653 | | | *WRi_006860* | | ankyrin repeat domain protein | | | | |
| 750749 | 753349 | | | *WRi_006870* | | ankyrin repeat domain protein | | | | |
| 754223 | 755143 | | | *WRi_006880* | | patatin family protein | | | | |
| 755153 | 755371 | | | *WRi_006890* | | hypothetical protein | | | | |
| 755496 | 756032 | | | *WRi_006900* | | ankyrin repeat domain protein | | | | |
| 755998 | 756978 | | | *WRi_006910* | | tail protein D putative | | | | |
| **CNV Location** | | | **Copy number change** | | | | **Affected genomes** | | **Kolmogorov-Smirnov *P*-value** |  |
| 1345000-1347500 | | | 1 → 2 | | | | *w*Suz only | | 0.016 |  |
| **Gene start** | **Gene end** | | | **Gene ID** | | **Gene Description** | | | | |
| 1345028 | 1345492 | | | *WRi_012540* | | baseplate assembly protein V | | | | |
| 1345769 | 1347103 | | | *WRi_012560* | | transposase | | | | |
